# Updates from 2018: Being indexed in Embase, becoming an affiliated journal of the World Federation for Medical Education, implementing an optional open data policy, adopting principles of transparency and best practice in scholarly publishing, and appreciation to reviewers

**DOI:** 10.3352/jeehp.2018.15.36

**Published:** 2018-12-28

**Authors:** Sun Huh

**Affiliations:** Department of Parasitology and Institute of Medical Education, Hallym University College of Medicine, Chuncheon, Korea; The Catholic University of Korea, Korea

We are proud to announce that in May 2018, *Journal of Educational Evaluation for Health Professions* (JEEHP) was included in Embase, the invaluable biomedical literature database maintained by Elsevier, which provides over 32 million records published in over 8,300 indexed peer-reviewed journals. This represents another opportunity for the journal to be disseminated and searched by biomedical researchers from all over the world. Although JEEHP has been indexed in MEDLINE since March 2016, it was included in Embase in May 2018. The rigorous review process for being indexed in Embase provides further evidence of the journal’s high quality and brand.

Although JEEHP has been an affiliated journal of the World Federation for Medical Education since September 2016 [1], this affiliation was not announced for a long time. There are 4 other affiliated journals: *Fundación Educacion Médica, Medical Education, Medical Teacher*, and *South-East Asian Journal of Medical Education*. The World Federation for Medical Education Conference 2019 will be held at the Grand Walkerhill Seoul in Seoul, Korea from April 7 to 10, 2019 (http://www.wfme2019.org/). I hope to invite the authors who present at this conference to publish their results in JEEHP.

In April 2016, we adopted an open data policy [2]. For 3 years, it has been well-maintained [3]; however, cases of withdrawal due to mandatory data deposition occurred frequently. Those cases involved students’ response files produced during an examination. Some institutions do not allow their faculty members to disseminate those data even if individual identifiers are removed. Therefore, we decided to change from a mandatory open data policy to an optional open data policy, in which authors come to an agreement with the JEEHP editorship about the sharing of raw data if examinees’ responses are included.

In January 2018, the third version of the ‘Principles of transparency and best practice in scholarly publishing’ was announced by the Committee on Publication Ethics, the Directory of Open Access Journals, the Open Access Scholarly Publishers Association, and the World Association of Medical Editors. It consists of 16 items that should be announced and adhered to by scholarly journal editors and publishers [4]. A compliance checklist of these 16 items was posted on the journal’s homepage on July 26, 2018. We will do our best to follow these best practice guidelines. The journal’s compliance with the best practice guidelines is not only a prerequisite to be a MEDLINE journal, but also a condition for continuing inclusion in PubMed Central.

This year, I invited a number of reviewers to review submissions to JEEHP. Some of them accepted the review request and provided invaluable comments that contributed to the better presentation of study results. Without their help, it would not be possible to publish this small, but unique, journal. I understand very well that they were all very busy, but they nonetheless shared their time to contribute to advances in educational evaluation for health professions. I appreciate them from the bottom of my heart. The reviewers for the 2018 issue are listed below:

Abdolghani Abdollahimohammad, Katharina Brandl, Su-Jin Chae, Lap Ki Chan, Cheol-Woon Chung, Derek Clewley, Fabrizi Consorti, Lionel Di Marco, Kyung (Chris) T. Han, Geum-Hee Jeong, Karen Huhn, Yera Hur, Oscar Jerez, Sun Hee Kang, Nayoung Kim, Yong Sung Kim, Na Jin Kim, Young-Min Kim, Sue Kim, A Ra Cho, Seock-Ho Kim, Kwang Hwan Kim, Sun Kim, Oh Young Kwon, Young Hwan Lee, Eun Young Lim, Nesreen El Mekawy, Younjae Oh, Cesar A. Orsini, Robin Parish, Janghee Park, Mee Young Park, Quan Pham, Amelia Richardson, Dong Gi Seo, Ji-Hyun Seo, Ravi Shankar, Luiz Troncon, Julie Youm, Hon Yuen, Pete Yunyongying, Gagani Athauda, Fabrizio Consorti, and Gregory Rose

We published 3 articles on computerized adaptive testing in this year’s issue introduction piece of SimulCAT, a software program developed for conducting CAT simulation studies [5]; post-hoc simulation study of computerized adaptive testing for the Korean Medical Licensing Examination [6]; and components of the item selection algorithm in computerized adaptive testing [7]. This kind of test has been implemented in high-stakes examinations for medical health professionals in the United States, including licensing examinations for nurses, pharmacists, clinical pathologists, and emergency medical technicians. One article described the 3 components of the item selection algorithm in computerized adaptive testing: test content balancing, the item selection criterion, and item exposure control. A variety of methods were explained [7]. This topic may be somewhat difficult for instructors in the medical and health fields, but it is possible that this form of testing will expand to Asian countries. Therefore, it is necessary to understand the basic concept of item response theory and computerized adaptive testing [8].

In the New Year, I hope to receive and publish more manuscripts with complete adherence to the journal’s style and format with interesting topics for instructors in the medical and health fields.
